# Explaining Intricate Morphometric Variability with Environmental Predictors: The Case of *Globularia cordifolia* Species Complex

**DOI:** 10.3390/plants9030314

**Published:** 2020-03-03

**Authors:** Michele Innangi, Maja Friščić, Kroata Hazler Pilepić, Tiziana Danise, Fabio Conti, Fabrizio Bartolucci, Antonietta Fioretto, Lorenzo Peruzzi

**Affiliations:** 1 Department of Environmental, Biological, Pharmaceutical Sciences and Technologies, University of Campania “Luigi Vanvitelli”, Via Vivaldi 43, 81100 Caserta, Italy; tiziana.danise@unicampania.it (T.D.); antonietta.fioretto@unicampania.it (A.F.); 2 Department of Pharmaceutical Botany, Faculty of Pharmacy and Biochemistry, University of Zagreb, Schrottova 39, 10000 Zagreb, Croatia; mfriscic@pharma.hr (M.F.); khazler@pharma.hr (K.H.P.); 3 Floristic Research Center of the Apennines, University of Camerino—Gran Sasso-Laga National Park, San Colombo, 67021 Barisciano (L’Aquiila), Italy; fabio.conti@unicam.it (F.C.); fabrizio.bartolucci@unicam.it (F.B.); 4 Department of Biology—Botany Unit, University of Pisa, Via Derna 11, 56126 Pisa, Italy; lorenzo.peruzzi@unipi.it

**Keywords:** *Globularia* section *Empetron*, *Globularia cordifolia*, classical morphometrics, geometric morphometrics, morphological variation, ecological statistics, geographical predictors, bioclimatic predictors

## Abstract

*Globularia* is a genus of small evergreen and perennial shrubs that are widespread in Europe. *Globularia* section *Empetron* includes a group of three species, *G. cordifolia*, *G. meridionalis*, and *G. neapolitana*, that have been taxonomically disputed for more than 150 years. Many morphological features have been proposed to discriminate these species. Nevertheless, evidence from both past and recent literature suggest that these differences among species are not consistent. In order to shed new light in this long-disputed group, we investigated 10 populations of the *G. cordifolia* species complex with both classical and geometric morphometrics and used environmental predictors in multivariate regression to explain patterns of variation. Our results showed that bract area and calyx teeth length are correlated with solar radiation and annual precipitation, whereas leaf dry mass per unit area can be explained by temperature seasonality. Leaf shape can be explained by temperature seasonality as well, although with a lower amount of explanatory and predictive power. Despite a comparatively low sample size in terms of populations, our results were based on a large number of individuals and were supported by a robust statistical approach. We can conclude that differences among the three species of *Globularia* could be related to the combined effects of several ecological variables and might not have taxonomical value. Our novel approach provided an ecological interpretation on a species complex that makes up a continuum of forms within the environmental framework of the Mediterranean basin.

## 1. Introduction

*Globularia* L. is a genus of angiosperms composed mostly of small evergreen and perennial shrubs, recognizable by spherical inflorescences of blue-violet flowers [1,2,3,4]. The number of species belonging to this genus and their taxonomic status have been interpreted in several ways by the authors who have studied these plants over the years. Currently, 27 taxa are included in this genus [5], of which many are narrow endemics [1,6,7]. Recent molecular studies have demonstrated that *Globularia*, which was traditionally included in a separate family (Globulariaceae) along with *Poskea* Vatke, actually belongs to Plantaginaceae [8]. Otto Schwarz proposed the first extensive classification of *Globularia*, recognizing 22–25 taxa [3,9]. Subsequently, after years of discordant or incomplete taxonomy, the genus underwent taxonomic modifications by several authors [4,6,10]. In the current classification of the genus *Globularia*, eight sections are recognized: *G.* section *Lytanthus* (Wettst.) O.Schwarz, *G.* section *Polycephalium* O.Schwarz, *G.* section *Carradoria* (A.DC.) Wettst., *G.* section *Hellenion* O.Schwarz, *G.* section *Globularia* (Syn.: section *Aphyllanthes* O.Schwarz), *G.* section *Alypum* (Fisch.) O.Schwarz, *G.* section *Empetron* O.Schwarz, and *G.* section *Gymnocladium* O.Schwarz [4]. Molecular data have provided evidence for Miocene origin of *Globularia*, and the independent evolution during the Pleistocene in three European Alpine and two Mediterranean groups [11,12,13,14]. Accordingly, representatives of the genus are widespread in most of Europe. However, the highest concentration of taxa is found in Central and Southern Europe, Anatolia, Northern Africa, and Macaronesia [1,5]. Disjunct populations are also found in the Atlantic and Swedish islands and around the Volga [5]. Most species are adapted to dry and open habitats, often on calcareous soils, in meadows or on bare rocks [6].

**Figure 1:**
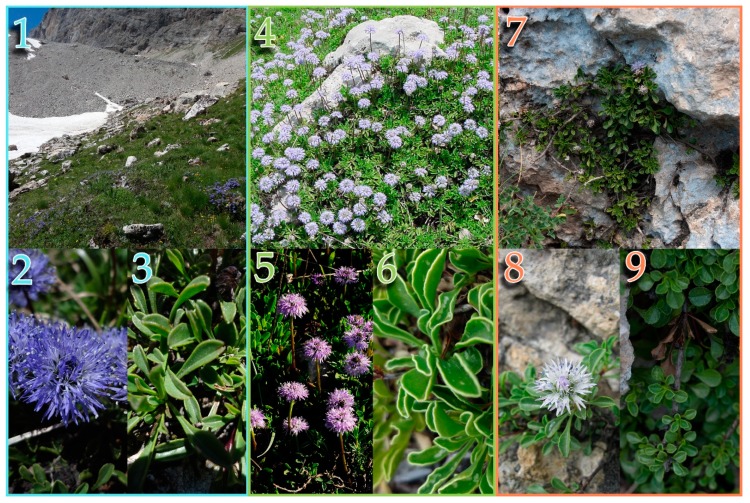
*Globularia cordifolia* species complex. Pictures 1–3 show habitat, flowers, and leaves of *G. cordifolia* from Val de Rhêmes, Vallée d’Aoste, Italy. Pictures 4–6 show *G. meridionalis* habitat and leaves from Timpa del Principe-Frascineto, Calabria, Italy, whereas picture 5 shows flowers from Fonte Vedice, Abruzzo, Italy. Pictures 7–9 show habitat, flowers, and leaves of *G. neapolitana* from Monte S. Angelo a Tre Pizzi, Campania, Italy. (Photos 1–4 and 6—L.P., 5—F.C., 7 and 9—M.I., and photo 8 was kindly granted by A. Izzo).

The typical life-forms of *Globularia* include hemicryptophytes and chamaephytes, with a primary root dominating the root system in most species during the whole life of the plant, with the exception of the *G. cordifolia* species complex (Figures 1 and 8) [4]. Stems are made of branches and stolons, along with flowering scapes, which often possess one to many bracts [4]. When in small number, such bracts on the flowering scape are known as scales [2]. True leaves, which are simple and mostly evergreen, can be arranged in several ways, but are generally sparse or fused in a basal rosette or on several bundles [4]. Flowers are collected in solitary capitula, with individual flowers clustered on a common receptacle. Such receptacles can be hairy, conical, cylindrical, or globose. The external flower bracts forming the envelope are more or less similar in shape to the internal ones, and both type of bracts often persist on the receptacle after flowering. Fruits are achenes enclosed in a persistent calyx, usually 1–1.5 × 0.5 mm [6,15].

Some *Globularia* species are well known for their medicinal use [16,17]. Accordingly, several species of *Globularia* have been phytochemically investigated, given their contents in iridoids and phenolic compounds with potential biological applications [18]. Conversely, in the last decades, the genus has been scarcely studied from a taxonomical point of view, with some exceptions [1,7,19,20,21]. During the years, some species have always been clearly recognizable from a taxonomic point of view (e.g., *Globularia alypum* L., *Globularia nudicaulis* L., or *Globularia incanescens* Viv.), whereas there has always been great confusion on *G. cordifolia* L. and allied species [6,21,22,23,24]. *Globularia* section *Empetron* includes *G. cordifolia*, *Globularia meridionalis* (Podp.) O.Schwarz, *Globularia repens* Lam., and the narrow endemic *Globularia neapolitana* O.Schwarz, which can all be found in Italy [2,4,25]. All of these species share most of their morphological character-states, being woody shrubs no taller than 25 cm, living in the same habitats, that is, calcareous rocks from sea level to high elevations (Figure 1) [2,6].

*Globularia cordifolia* shows the largest area of occurrence, from Turkey to Spain, whereas *G. repens* is limited to the western Mediterranean, from Spain to northern Italy [6]. *Globularia meridionalis* is restricted to the Balkans, from Greece and Bulgaria to Croatia, Slovenia, and Austria, and extends along the Italian Apennines [3]. In the southern Apennines, the narrow endemic *G. neapolitana* is known for some populations in the Sorrento-Amalfi peninsula and the Island of Capri [26,27,28], although some populations have been reported in the nearby Taburno-Camposauro complex in sympatry with *G. meridionalis* [29,30]. According to early karyological data, *G. meridionalis* is diploid with 2*n* = 16 chromosomes, although autopolyploids exist with 2*n* = 32 chromosomes, whereas *G. cordifolia* (2*n* = 32) is an allotetraploid allegedly originating from *G. repens* (2*n* = 16) and *G. meridionalis* [9,31]. However, according to Milletti [6], there is no evidence of true karyological differences between *G. cordifolia* and *G. meridionalis*, given that tetraploid level 2*n* = 32 is reported for both species, as confirmed also by other authors [22]. On the other hand, *G. repens* is diploid with 2*n* = 16 chromosomes [10], and *G. neapolitana* has been reported as tetraploid (2*n* = 32) or even 2*n* = 16 in some cases [23]. Thus, with the exception of *G. repens*, all species in *G.* section *Empetron* are, or can be, tetraploid; share the same habitats; and largely overlap in their distribution.

Concerning the morphological features that have been used to distinguish these species, Milletti [6] made an extensive survey of the different criteria used throughout the years, which can be summarized as (a) the general habitus of the plant, including its life-form and overall size; (b) the leaf size and its apex shape; (c) the scale number on the flowering scape; (d) the shape of the outer and inner bracts, including the presence and abundance of hairs; and (e) the calyx shape, with particular regard to the ratio between the teeth and the tube length. Pignatti [2] used a combination of the aforementioned features to circumscribe the species complex within *G.* section *Empetron*, but according to Milletti [6] there is too much variability in most of these features, with the exception of the calyx. According to Ravnik [21,22], populations from *G. cordifolia* species complex can be different from one another in the size and shape of their leaf blades, the serration of the leaf edge, the type of the flower calyx and corolla, and the shape and sharpness of the outer bracts. However, they never differ in all of these features, whereas different types of the same feature can, at the same time, be present in an individual plant. The same author suggested these differences to be consequences of polymorphisms and proposed to consider the sole taxon *G. cordifolia* [21,22].

Evidence from recent molecular data support the hypothesis that *G.* section *Empetron* is one of the latest branching lineages in the evolution of *Globularia*, and that *G. meridionalis, G. cordifolia*, and *G. neapolitana* are close to *G. repens* [1,5]. According to Milletti [6], such large variability could be explained by the wide area of occurrence of the species and their habitat fragmentation, noting a large polymorphism and quoting Willkomm [32] for the explanation, “*Hic speciei typus per innumeras formas intermedias transit in varietatem”* (English: “*This type of species passes from variety to variety through countless intermediate forms”*). Admittedly, mechanisms of speciation are still poorly understood in *Globularia*, and fine-scale distributional, population genetic, morphological, and reproductive data are needed to further clarify the evolutionary history of these plants [5].

Yet, there is a large amount of recent literature exploring morphological variations in plants as dependent on environmental factors. For instance, sea daffodils (*Pancratium maritimum* L.) vary their morphological features according to the bioclimatic variables to which they are exposed, including precipitation, average annual temperatures, and strong irradiance [33]. Subtler climate effects were observed in *Pinus flexilis* E.James, where there was no macroscopic variation in morphology on a large elevation gradient (1600–3300 m), yet stomatal density was inversely correlated with elevation [34]. Climate has been shown to have strong potential effects on flower morphology as well [35]. Undoubtedly, the availability of global-scale bioclimatic variables [36] and the introduction of novel statistical techniques can help to shed new light to ecological and biological phenomena [37]. Yet, the study of morphology remains a key-instrument in plant systematics and ecology, both with traditional approaches [38,39,40] or with modern geometric morphometrics [41,42,43].

**Figure 2:**
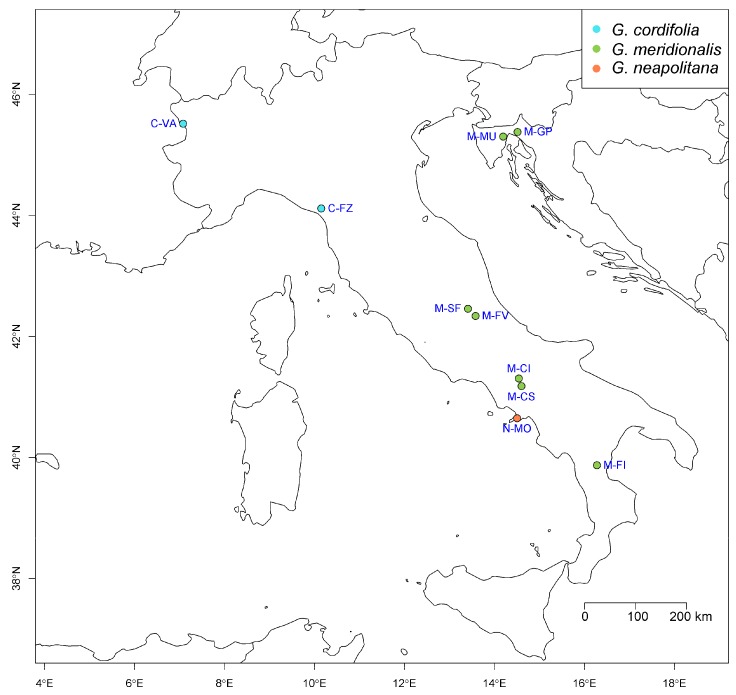
Distribution of the sampled populations of *G. cordifolia*, *G. meridionalis*, and *G. neapolitana*. For details about the single populations, refer to Table 3.

Thus, in our research, we tested the hypothesis that the variability in shape and size observed in *G. cordifolia* species complex could be explained by the impact of bioclimatic variables. In order to do so, we used both classical and geometric morphometrics, by extracting principal components (PCs) from multivariate datasets and by using them as outcomes, as well as bioclimatic variables as predictors, in order to test the effect of climate on size and shape variability. Although our final outputs were based on a comparatively small sample size (10 populations; Figure 2 and Table 3), the data were derived from a large number of individuals and organs per populations, and were based on robust, cross-validated statistical models.

## 2. Results

### 2.1. Classical Morphometrics

Results from classical morphometric analysis for all weakly correlated variables used in the Principal component analysis (PCA) can be seen in Table 1, whereas all other variables are available in Supplement 1 and Supplement 2. Leaf per mass area (LMA) was not strongly different among populations, with a mean value of 199.68 ± 3.79 g/m^2^. Maximum values were reached in *G. meridionalis* (M-GP) and minimum in *G. neapolitana* (N-MO). No remarkable difference emerged among the studied species, with the two populations of *G. cordifolia* comparable to the seven *G. meridionalis,* as well as to the single population of *G. neapolitana*. Data concerning the calyx showed moderate amount of variability. As for tube length, highest values (≥1.8 mm) were reached in several populations (C-FZ, M-CS, N-MO, and M-FI) that belong to all studied species, whereas low values were found in M-MU (1.47 ± 0.07 mm). Additionally, on one hand, the length of the calyx teeth varied from 2.33 ± 0.11 mm in C-VA (*G. cordifolia*) to 1.82 ± 0.06 in M-CS (*G. meridionalis*). On the other hand, there was no pattern among species, with populations as distant as almost 1000 km (C-VA vs. M-FI, see Figure 2) showing no significant difference. Thus, the ratio between the lengths of the calyx teeth and tube also showed little variability, with a range of 1.0–1.5. Once again, there was no noticeable pattern among populations and within species, with a few populations showing calyx teeth clearly longer than the tube (C-VA, M-MU, M-FV, Supplement 1). Data for the outer bracts showed an even smaller degree of variability. Accordingly, all populations shared similar values of outer bract area, with a mean value of 3.95 ± 0.14 mm^2^. Similarly, inner bract length showed little variability, with significant differences only between M-GP and M-FI, with a similar pattern for inner bract area, which was on average 2.84 ± 0.08 mm^2^. Significant differences for inner bract area were found only between N-MO and M-FV, whereas all other values were intermediate. Noticeably, when considering variability at a species level rather than at population level, the only variable that showed significant differences was LMA, which was higher in *G. meridionalis* (208.74 ± 4.51 g m^−2^) compared to both *G. cordifolia* and *G. neapolitana* (Table 1).

**Figure 3:**
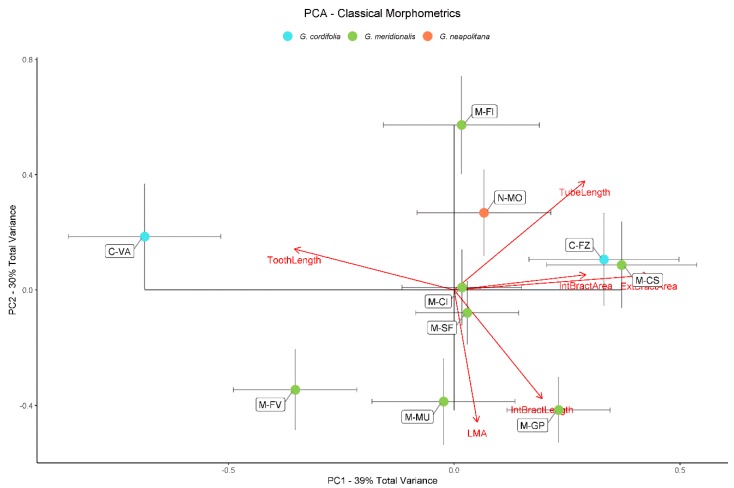
Principal component analysis for classical morphometric data. Red arrows represent loadings along principal component (PC)1 and PC2. Each population (see Table 1) is represented by mean ± standard error of the mean of the scores along PC1 and PC2 in a different color according to species.

Principal component analysis (PCA) for classical morphometric data showed that 69% of total variance could be explained by the first two PCs (Figure 3). Accordingly, 39% of total variance was explained by PC1, highlighting that outer and inner bracts showed a positive correlation separating, on the one side, *G. meridionalis* and *G. cordifolia* populations (M-CS and C-FZ), whereas, on the other side, teeth length separated C-VA (*G. cordifolia*) and, to a lesser degree, M-FV (*G. meridionalis*). According to the directions of PC2, which explained 30% of the total variance, some populations were clearly separated by tube length (M-FI), and both LMA and inner bract lengths (M-MU, M-GP, and M-FV). We also explored PC3 (Supplement 3) that explained 17% of total variance, but without any relevant discrimination between populations and/or species.

**Figure 4:**
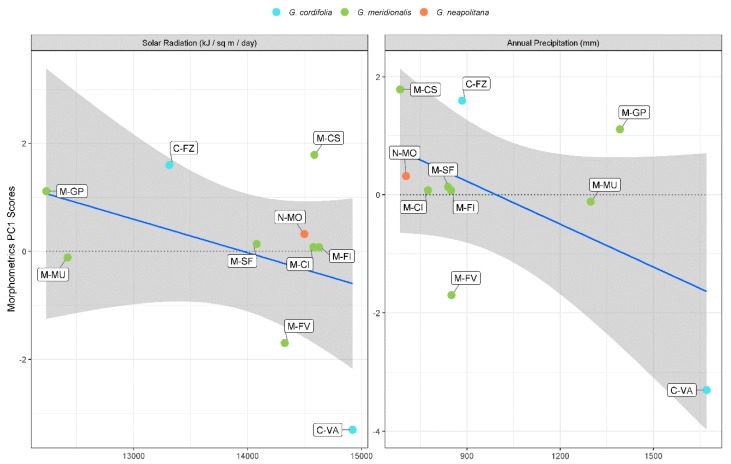
Scatterplot between classical morphometric PC1 scores and significant environmental predictors.

**Figure 5:**
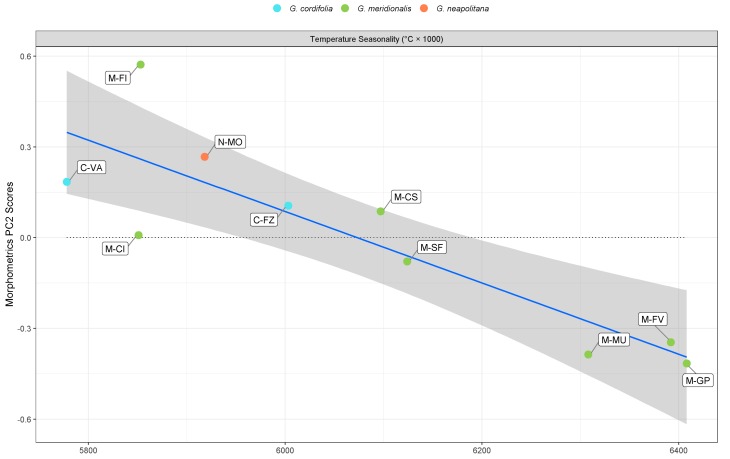
Scatterplot between classical morphometric PC2 scores and temperature seasonality.

Modelling PC1 for classical morphometrics with environmental predictors showed a good fit, with high values of both explanatory and predictive power after cross-validation (*p* < 0.050) (Table 2). Significant predictors were solar radiation and annual precipitation. Noticeably, there was an inverse trend in both predictors and PC1 scores (Figure 4). Solar radiation was ≥14,000 kJ/m^2^ /day for most populations, whereas only three populations (C-FZ, M-MU, and M-GP) were below this threshold. The PCA scores for C-VA, M-FV, and M-CS were outliers when correlated with solar radiation. As for annual precipitation, a clearer separation was shown by most population growing below 900 mm, with the exception of M-MU, M-GP, and C-VA. M-FV and M-GP were outlying in the correlation with annual precipitation. The model for PC2 showed a higher amount of both explanatory and predictive power after cross-validation for a highly significant model (*p* < 0.010, Table 2). Temperature seasonality was the only significant predictor, which neatly separated populations such as M-MU, M-FV, and M-GP from all other populations (Figure 5). With the exception of M-FI and M-CI, all populations showed a clear inverse morphometric trend on PC2 with the increase of temperature seasonality. Temperature seasonality above 6.2 °C correlated with increase in LMA and inner bract length, whereas the opposite was true for calyx teeth length. Both models for PC1 and PC2 in classical morphometrics showed high values of concordance correlation coefficient (CCC) and low values of normalized mean absolute error (nMAE), supporting model reliability despite the comparatively low population sample size.

### 2.2. Geometric Morphometrics

**Figure 6:**
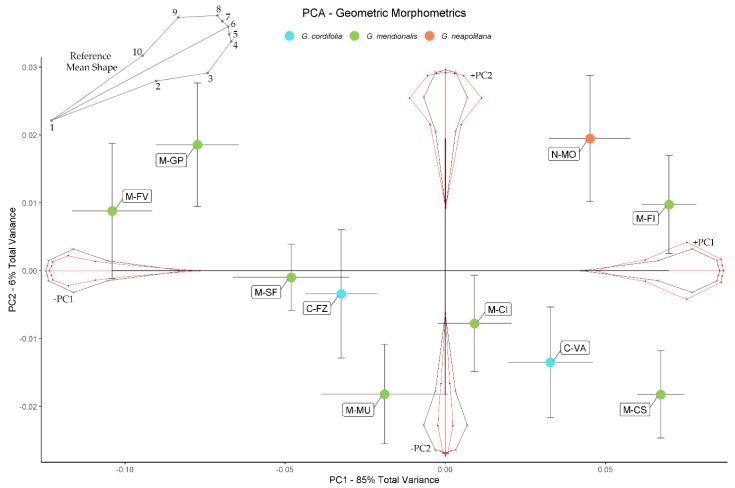
Principal component analysis for geometric morphometric data. Shape variations on each PC and according to the PC sign are shown as exaggerated ±20% deformations (red) from the reference mean shape (black). A reference mean shape with the position of landmark and semi-landmarks is shown in the upper left corner. Each population is represented by mean ± standard error of the mean of the scores along PC1 and PC2 in different color according to species.

The multiple regression of shape on size was highly significant (*p* = 0.002), and thus allometry was strongly present. Once size-corrected, results for PCA in geometric morphometrics can be seen in Figure 6. Almost all variance (91%) is summarized in the first two PCs, with PC1 explaining 85% of total variance and PC2 explaining 6%. The relative changes in shape according to the direction of PC1 show that populations from all species (N-MO, M-FI, M-CS, C-VA, and M-CI) are distinguished from all other populations by leaf shape, which was broader than the average shape, whereas leaves from other populations were more elliptical, with strong variations on the position of landmarks 2–10 and 3–9. Although explaining little variance, PC2 showed shovel-shaped or narrow leaves in positive and negative directions, respectively. Leaf apex was also affected by PC2, with rounder vs. pointed tips in the two directions of the axis, without evidence of other apex morphologies (e.g., rounded vs. three-toothed, see Figure 9).

**Figure 7:**
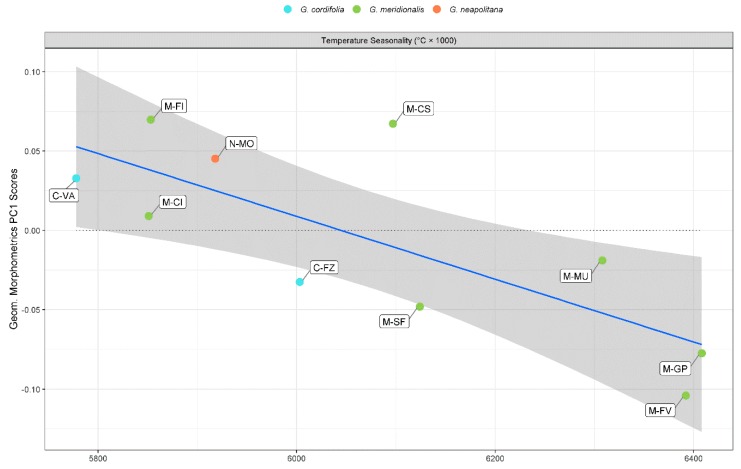
Scatterplot between geometric morphometric PC1 scores and temperature seasonality.

In line with classical morphometrics, temperature seasonality was the most significant predictor in the model for PC1 in geometric morphometrics (Table 2 and Figure 7). The model, despite a comparatively lower explanatory and predictive power than classical morphometrics, proved to be significant (*p* < 0.050). Leaves from some populations (e.g., M-GP and M-FV) showed a difference in shape from other populations (e.g., C-VA and M-FI), coherent with the reduction in temperature seasonality. Thus, larger temperature seasonality (≥6.2 °C) is reflected in narrower leaves, whereas smaller temperature seasonality (≤ 6.0 °C) broadens leaf shape. On the contrary, PC2 model in geometric morphometrics was not significant (Table 2). The only variable included in the model was the mean temperature of driest quarter, showing a weak but not significant positive trend with PC2 (Supplement 4). The values of CCC and nMAE for the PC1 geometric morphometric model confirmed its reliability after cross-validation.

## 3. Discussion

Our morphometric approach confirmed the great variability in *G. cordifolia* species complex, both in size and shape. A set of morphometric features that would reliably discriminate *G. cordifolia*, *G. meridionalis*, and *G. neapolitana* was not observed, supporting the results from earlier morphological investigations of the same species [6,21,22]. All morphometric variables, when considered at a population level, did not show significant differences, with the exception of LMA. Pignatti [2] suggested that the key diagnostic characteristic is the ratio between calyx teeth and tube, with the tube being much longer in *G. cordifolia* compared to that in *G. meridionalis*, in which teeth and tube should be sub-equal, whereas in *G. neapolitana,* teeth are longer than the tube. Our results did not confirm such a pattern, given that teeth are clearly longer than the tube only for *G. cordifolia* (C-VA) and *G. meridionalis* (M-FV, M-MU, M-CI), whereas all other populations showed teeth slightly longer than, or sub-equal to tubes (C-FZ, M-CS, and M-FI). Similarly, Milletti [6] found that the calyx teeth were always longer than the tube, and rarely sub-equal. Concerning the separation of *G. cordifolia* from *G. meridionalis*, a distinction on outer bracts was also proposed, specifically on the presence of hairs and the shape of the bracts characterized by the maximum width positioned at the base or at the center of the bract [2]. We did observe the presence of hairs in some populations of *G. meridionalis* (see Supplement 2), yet with a great degree of variability in abundance, and we agree with Milletti [6] that this character can be deemed unreliable. Outer bracts were reported as strongly mucronate in *G. neapolitana* [2], a feature that we did notice in most specimens from N-MO (see Supplement 2), although there was no difference in their size compared with those of *G. cordifolia* and *G. meridionalis*. Milletti [6] observed that the only true morphological differences existing between *G. repens* and *G. cordifolia* concern the leaves not exceeding 2 × 0.5 cm, always with entire apex, in the former. Conversely, *G. meridionalis* was considered a heterotypic synonym of *G. cordifolia*, whereas *G. neapolitana* was retained as a subspecies of *G. cordifolia* [6,23], being distinguished by leaf shape, that is, lamina from spatulate-obovate to suborbicular with crenate-undulate margin vs. lamina from oblanceolate-obovate to spatulate-cuneiform with entire margin [6]. *Globularia neapolitana* was also reported as bearing nude flowering scapes [6].

On the basis of such a confused taxonomic picture, our novel approach shed new light on the morphological variability in *G. cordifolia* species complex, showing that ecological predictors could explain both size and shape, revealing an apparent lack of taxonomical differences among species. Most of the variation in size, involving leaf and bract morphological variables, could be explained by a combination of solar radiation, temperature seasonality, and annual precipitation. Among the several available environmental predictors, we found that elevation was not significant in any model. Accordingly, previous evidence showed that elevation could not influence morphology in *G. cordifolia* [6], although there has been no in-depth study on *Globularia* morphology on elevation gradients. The evidence from literature highlighted that elevation might cause a variation in a clinal pattern, as was observed with *Penstemon* sp. pl. [44], or even no variation on strong altitudinal gradients, as observed in *Sesleria rigida* Heuff. ex Rchb. [45]. In our case, variation on classical morphometric PC1 was shown to be correlated with a combination of solar radiation and annual precipitation, with noticeable changes in flower and receptacle variables. Solar radiation, especially UV-B radiation, can trigger a broad range of responses in plants at the molecular, cellular, and organism level, including the structure of the inflorescences [46,47]. In bromeliads from xeric environments, variations in the shape of the rosette, leaf color, and size of the leaf sheath and blade were shown to be correlated with solar radiation [48]. As for annual precipitation, anatomical differentiation of populations of *S. rigida* were found to be significantly correlated with annual precipitation and the precipitation of the wettest month, whereas temperatures were not significant [45]. Conversely, there is a general consensus that mean annual temperatures are significantly more strongly correlated with plant traits than mean annual precipitation, although such evidence can be biased due to the weak link between mean annual precipitation and the availability of water to plants [49].

We presented, for the first time, extensive LMA measurements for *G. cordifolia* species complex. Pierce et al. [50] reported specific leaf area only for *G. cordifolia* as 6.3 mm^2^ mg^−1^. This value corresponds to LMA value of 158.7 g/m^2^, which is in line with our measurements. From a life-form perspective, LMA values like those we have found in our samples are reported as typical for evergreen shrubs, although they are overlapping also with evergreen gymnosperms and succulent plants [51]. From an ecological point of view, values of 200 g/m^2^ are typical for shrubland or desert species [51]. These data confirm the known ecology and evolutionary history of *Globularia* section *Empetron* as adapted to arid rocky xeric environments [1,5,12], but do not contribute to their taxonomy. LMA was shown to be significantly higher for *G. meridionalis* than the other two species, but LMA was also the driving variable on PC2 for classical morphometrics and PC1 for geometric morphometrics which, in turn, were correlated with temperature seasonality. Temperature seasonality is a measure of temperature change over the course of the year and it is computed as standard deviation of the 12 mean monthly temperature [52]. This variable has been shown to be an important ecological predictor for the distribution of many plant species, either rare or widespread [53,54,55,56,57]. This predictor clearly helped us to distinguish some populations (M-GP, M-MU, and M-FV) that are above 6.2 °C temperature seasonality, exhibiting the highest values of most morphological variables, as well as also explaining most of the variation in shape. Comparing the values of temperature seasonality with LMA, M-MU, M-SF, and M-FV showed LMA values above 200 g/m^2^ and up to 260 g/m^2^ for M-GP. According to ecological literature, these LMA values indicate that either drought, nutrient limitation, or both can have a limitative effect on plant growth [51], which can give insights to a morphological adaptation to specific stresses.

We cannot be completely sure on how these morphological variations can be beneficial for the life of *Globularia* populations as a response of ecophysiology. Undoubtedly, plants can show a surprising degree of morphological plasticity at either small or large scale. As a matter of fact, in two populations of *Prunus serotina* Ehrh., one from a xeric and one from a mesic environment, sun leaves from the xeric population had greater thickness, specific mass, and guard cell length than the sun leaves from the mesic population, yet no morphological difference was found in shade plants in either population [58]. Plants that have evolved in arid environments, such as *G. cordifolia*, can show morphological changes to environmental modifications at a comparatively fast rate. For instance, two desert shrubs from Central Asia responded to recent increase in precipitation both in their morphology and physiology [59].

Changes in calyx and receptacle size could also reflect changes in pollinators, given that coevolution with pollinators can generate within-species geographic variation in the morphology of plant species, eventually leading to plant speciation [60]. Pollinators can be “drivers” of variation, such as in the case of *Arum maculatum* L., a widespread species with a specialized pollination system [61]. In this plant and its pollinators (two species of flies), a geographically structured variability in pollinators was found, with increasing proportion of one species with higher annual precipitation and lower precipitation in the warmest trimester, two features typical of the Mediterranean zone [61]. *Globularia* flowers are bilabiate and protogynous, and observed pollinators have been butterflies, bees, beetles, and syrphids [6,62,63], although detailed study on pollinators in this genus, especially in *G. cordifolia* species complex or along elevation gradients, are scarce and outdated [64]. Thus, it cannot be ruled out that the trends in calyx and receptacle morphology can be a consequence of a shift in pollinators on climatic gradients.

Our study on size was accompanied by a geometric morphometrics approach on leaves. Our test for allometry showed that there was a strong effect of size onto shape. Allometry can be a pivotal component of shape variation, and generalized procrustes analysis (GPA) removes isometric effects of size on shape, but not allometric effects [65]. Thus, our approach of performing shape analysis on size-corrected shapes allowed us to interpret only the variation on shape once allometric affects have been taken into account [66,67]. Given the lower amount of variation of modelling with PC1 of geometric morphometrics, the variation of shape was smaller than size and less correlated with ecological predictors. We found that temperature seasonality can explain most of the variations in shape, in the same way as it explained variation in PC2 for classical morphometrics. Leaf shape variations have been shown to be a functional response to altitude and longitude at regional scales rather than to temperature-related factors such as latitude [68]. Environmental gradients in shapes, with special regard to elevation, were also found in *Sophora davidii* Franch., providing strong evidence that variations in morphological and genetic parameters reflect morphological and genetic adaptation to native habitats, highlighting ecological and evolutionary consequences along altitudinal gradients of mountainous ecosystems [69]. The shape of the leaf apex was widely indicated in the past as diagnostic [2,15,70]. Nevertheless, the shape of the leaf apex (rounded vs. mucronate vs. three-toothed) has been considered a largely inconstant character, greatly varying among and within populations (see Figure 9) [6,21,22]. Ravnik [21,22] also reported that different leaf shapes, that, according to Schwarz [3] are characteristic for *G. cordifolia* or *G. meridionalis*, can be present in a single plant at the same time. We found no relevant variation in the leaf-apex landmarks in our shape analysis.

Despite a relatively small sample size compared to the area of occurrence of the studied plants, which might limit the breadth of this research, we provided evidence that the variability in size and shape within *G. cordifolia* species complex could be connected to environmental factors. Accordingly, we did not observe any reliable morphological pattern that would support the current classification into different species, albeit our research was ecologically oriented and not a taxonomic study. Admittedly, the observed absence of morphological evidence that would support current classification *G. cordifolia* species complex might simply be a consequence of low statistical power due to our insufficient sample size, along with the underrepresentation of *G. cordifolia* (two samples) and the narrow endemic *G. neapolitana* (one sample). Nevertheless, our analysis was supported by an underlying large sample size per individuals as well as robust evidence from cross-validation of the models.

Moreover, it should be noted that lack of characteristics that would support the separation of *G. cordifolia* and *G. meridionalis* into different species was recently recorded by Friščić [71], who studied different aspects of *Globularia* species from Croatia. On the basis of molecular [1,5], phytochemical [18,72,73], and karyological data [6,22,71], as well as previous morphological data [6,21,22]*, G. meridionalis* formed a continuum with *G. cordifolia* and might be considered without any taxonomical value as indicated also by earlier studies of plant material from Italy, Slovenia, Croatia, Bosnia and Herzegovina, Serbia, and Macedonia [6,21,22,23,24]. Concerning *G. neapolitana*, the single population that we sampled (N-MO) falls within the same morphological pattern, and might therefore be included as a heterotypic synonym of *G. cordifolia*. On the basis of our evidence, the reported sympatry between *G. meridionalis* and *G. neapolitana* [30] would be unrealistic. According to molecular data, *G. neapolitana* forms a single lineage with *G. cordifolia*, with the only difference that possible ancestral ranges for *G. cordifolia* should be from the Circumboreal Region, whereas *G. neapolitana* belongs to the Mediterranean basin [5]. Nevertheless, literature data suggest that a population referred as *G. neapolitana* from the island of Capri, given its isolation and low-elevation, shows morphological differences substantiating its taxonomic position as a distinct unit [6]. Such peculiarity is confirmed by anomalies in the chromosome counts and by cultivation experiments, where plants retained their morphology, suggesting a genetic stabilization of characters [6,23]. A herbarium sheet by G. Gussone dating back to 1808 is present in the Herbarium Neapolitanum (NAP!). The original label refers to *G. bellidifolia* Ten. with a vague geographical reference to Capri, with no indication of precise toponyms. The sheet was later revised by M. Ricciardi in 1976, who identified the samples as *G. neapolitana*. The conservation status of the herbarium sheet, the low number of organs, and the impossibility of evaluating clone sampling prevented us from considering this herbarium sheet in our analysis. Despite field research, we were unable to retrieve any *Globularia* populations in Capri, but we cannot exclude that, at least in this population, there could be a patroendemic systematic unit [74].

Given its ploidy level, recent origin, and distribution, *G. cordifolia* is likely a result of glaciations [12]. Polyploid species are favored under stressful environmental conditions [75], and patterns of morphological clines within and among plant species are known in the Mediterranean basin, as in the case of *Pinguicula* [76], *Soldanella* [77]*, Fritillaria* [78]*,* and other mountain-Mediterranean taxa, including *Globularia* sp. pl. from other sections besides *G.* section *Empetron* [5,13,14]. Although there is a need for a larger scale genetic and morphometric analysis in *G. cordifolia* species complex [1], along with an in-depth cultivation study to assess if there is a potential genetic basis for plant traits [79] and/or any effect of soil, our morphological analysis, along with ecological statistics, helped to bring about a new perspective this puzzling group of species.

## 4. Conclusions

According to our results, the variability in size and shape within *G. cordifolia* species complex could be explained by environmental variables, such as temperature seasonality. A reliable pattern that would go in favor of the classification into three distinguished species, namely, *G. cordifolia*, *G. meridionalis,* and *G. neapolitana*, was not observed in this study, although definitive taxonomic implications fall behind the scope of this research. Acknowledging the relatively small sample size of populations and the fact that samples were collected only from a limited geographical area (Italy and Croatia), it would be desirable to further expand the study on populations from a wider distribution area and verify, not only by means of morphometrics but also by DNA-based analysis including common garden experiments, whether the current classification into different species is justifiable or not. Nevertheless, we provided evidence that the environment might have been the main driver behind the large morphological variability in *G. cordifolia* species complex, which has confused taxonomists for more than 150 years.

## 5. Materials and Methods

### 5.1. Sample Collection

We sampled 10 different populations of *G. cordifolia* species complex (Figure 1) from a wide part of its area of occurrence (Table 3 and Figure 2), with a minimum distance between populations of 15 km (M-CS vs. M-CI) and a maximum of 979 km (C-VA vs. M-FI, see Figure 2). The identification of the populations, according to current nomenclature and known distributions, was performed following the methods in [2,3]. We focused on *G. cordifolia*, *G. meridionalis*, and *G. neapolitana*, given that *G. repens* had been previously shown to be clearly separated from other species of *G.* section *Empetron* on many levels [1,5,6]. Between June and October 2017, 11-16 specimens per population were collected. Individual plants were chosen as being at least 5 m apart, in order to reduce the risk of clone-sampling, and a branch with its flowering scapes was cut from the plant. All samples were dried and kept on herbarium sheets until processing. A high-resolution digital version of voucher specimens is presented in Supplement 5. At the sampling time, most populations were bearing fruits and not flowers. However, calyces persist after flowering, whereas corollas have been shown to have no diagnostic power [6], especially considering that tentative differences can be appreciated only on fresh specimens but not on dried ones [2]. Similarly, bracts along the flowering scape (i.e., scales) were deemed unreliable both in their number and their morphology according to the literature [4,6]. According to the conservation status of the different specimens within the population and the availability of intact organs, we performed measurements on 674 leaves (i.e., between 4 and 6 leaves per specimen), 351 calyces (i.e., 1–6 calyces per specimen), 86 external bracts (i.e., 1–3 bracts per specimen), and 196 internal bracts (i.e., 1–4 bracts per specimens). All organs were randomly chosen on each specimen in order to account for their variability, avoiding organs that were damaged or clearly under development. Nevertheless, the means per individuals within each population, followed by grand mean per population, were used for the statistical analyses. A complete imagery and data collection from all individuals is available as Supplement 2.

### 5.2. Classical Morphometrics

**Figure 8:**
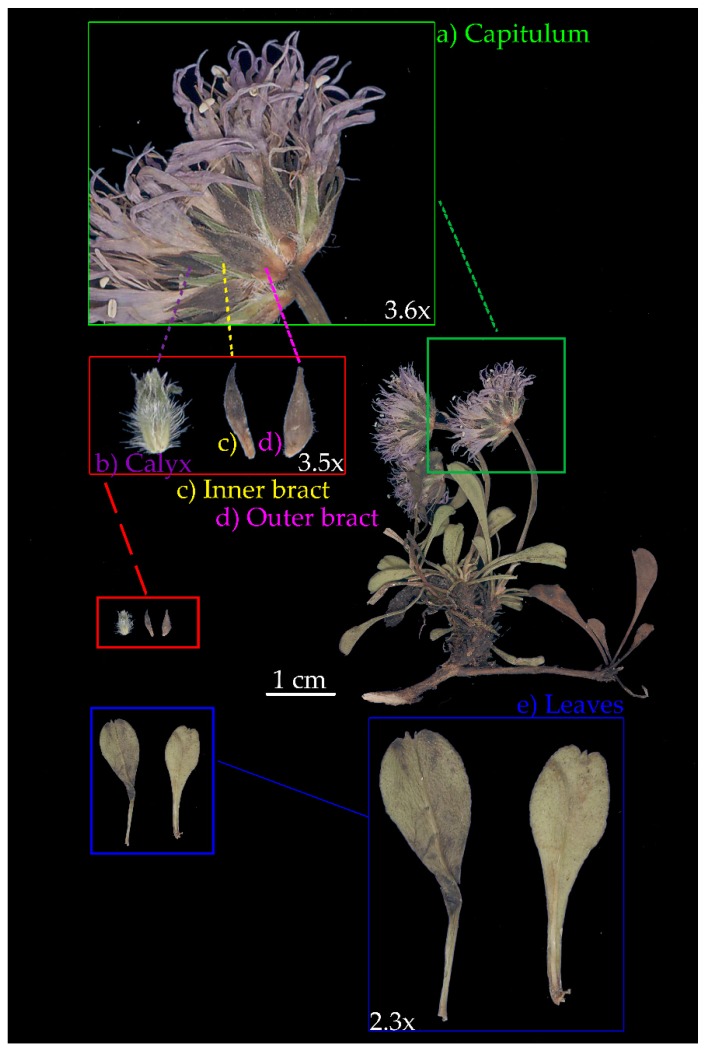
Details of a branch from *Globularia cordifolia* (Val de Rhêmes, Vallée d’Aoste, Italy). The inflorescence (**a**) (capitulum) is shown in the green box at 3.6× magnification. Details of the calyx (**b**), inner (**c**), and outer bracts (**d**) are shown in the red box at 3.5× magnification. Leaves (**e**) are shown in the blue box at 2.3× magnification.

Individual specimens per population were scanned at high resolution (1200 dpi) with a metric reference (see also Supplement 2). An example specimen, with all measured organs, is shown in Figure 8. According to the literature, we collected morphometric data on calyces (Figure 8b), inner and outer bracts (Figure 8c–d), and leaves (Figure 8e) [2,6]. In detail, we measured 17 variables, namely, (a) area (mm^2^), length (mm), width (mm), length/width ratio, dry weight (mg), and leaf dry mass per unit area (LMA; g/m^2^) for leaves; (b) area (mm^2^), length (mm), width (mm), and length/width ratio for inner bracts; (c) area (mm^2^), length (mm), width (mm), and length/width ratio for outer bracts; and (d) tube length (mm), teeth length (mm), and teeth/tube length ratio for calyces. All morphometric measurements were performed using the tps series of software (https://life.bio.sunysb.edu/ee/rohlf/software.html) [80], with the exception of leaf dry weight, hich was measured using an analytical balance (precision 0.001 mg). Leaf dry mass per unit area (LMA) was calculated according to [81].

### 5.3. Geometric Morphometrics

**Figure 9:**
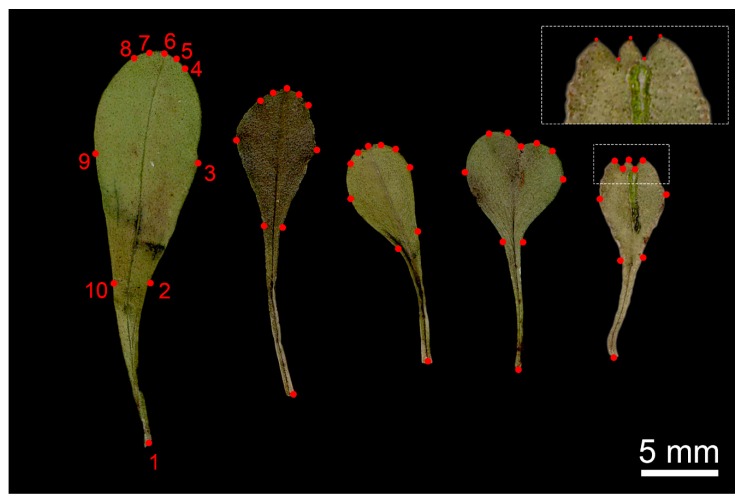
Morphometric gradient in leaves from *G. meridionalis* with landmark configuration. From left to right, a gradient in size and shape is shown, especially in the leaf apex from rounded to three-toothed. Landmark description: 1 and 6, petiole and leaf tips; 2 and 10, points where the blade begins to expand; 3 and 9, points of maximum width; 4 and 5, and 7 and 8, semilandmarks to describe the shape of the leaf apex (details are shown in the box).

Leaves were also analyzed using a geometric morphometric approach. We used generalized procrustes analysis (GPA) as a superimposition method, considering the objects as symmetric [82]. GPA is an established procedure for analysis of 2D and 3D shapes, which has been widely applied to plants in the last years [83,84,85,86,87,88]. Although objects such as leaves can be analyzed also by means of elliptical Fourier analysis [89], we decided that a landmark approach was more suited to detect possible subtle changes with potential diagnostic value, such as those considering the morphology of leaf apex. We opted for a configuration of 10 landmarks, designed to represent the putative diagnostic features that were reported during the years (Figure 9). Landmarks 1 and 6 represent petiole and leaf tips, respectively; 2 and 10 are the points where the blade begins to expand; 3 and 9 represent maximum width; and points 4 and 5, and 7 and 8 are semi-landmarks [90] to describe the shape of the leaf apex, which has been reported as diagnostic by several authors [2,15,70]. Please note the detail in Figure 9 that depicts the configuration of the latter landmarks, designed to detect the large variability in leaf apex from rounded to three-toothed. Landmarks were digitized using software from the tps series [80]. Digitizing was performed with tpsDig and repeated twice after two weeks on a random subsample of pictures in order to assess the error in digitizing, which was around 2–3%. After digitizing, GPA was performed in MorphoJ [91]. In order to account for allometric effects, we performed a multivariate regression of shape onto size using the natural logarithm of centroid size of every shape as an independent variable. Thus, the residuals of this regression can be considered as “size-corrected” shapes, permitting a better interpretation of the variations in shape once size has been taken into account [67,86].

### 5.4. Statistics and Modeling

All data were inspected for outliers by creating sub-datasets per population and performing principal component analysis (PCA). All specimens exceeding a 95% ellipse of the distribution in the scatterplot of the first two components were considered outliers and excluded from further analyses [86]. Given the large degree of collinearity in morphometrics [67], we reduced the number of variables in the classical morphometric dataset to weakly correlated variables, recursively excluding those that were strongly correlated (Pearson’s correlation coefficient ≥|0.85|). The procedure retained LMA, calyx tube and teeth lengths, outer bract area, and inner bract length and area. After this procedure, minimum correlation was *r* = 0.11 and maximum was *r* = −0.64. Differences among populations or species were tested by one-way ANOVA. Data were transformed by Box-Cox transformation with optimal lambda to ensure normality and homogeneity of variances [92]. Multiple comparisons were performed by Games–Howell *post-hoc* test (α = 0.05). On both classical and geometric morphometric datasets, we performed PCA. Scores for the first two PCs were extracted and used as dependent variables for ecological modelling [93,94,95,96]. Geographical and bioclimatic variables at 1 × 1 km spatial resolution were obtained from www.worldclim.org [36] and used as predictors in the modelling procedure. Predictors included latitude, longitude, elevation, solar radiation, wind speed, water vapor pressure, and 19 bioclimatic variables (bio_01–bio_19) derived from temperature and precipitation [52]. To avoid multicollinearity in the predictors, bioclimatic variables were selected using a variance inflation factor (VIF) approach by recursively dropping all variables with VIF ≥3 [55,56]. The procedure retained elevation, solar radiation, bio_04 (temperature seasonality), bio_09 (mean temperature of driest quarter), and bio_12 (annual precipitation). We tested the presence of spatial autocorrelation by means of Moran’s I coefficients [97,98], but we found no evidence of it in all cases. Subsequently, we fitted multiple linear regressions with Akaike Information Criterion based stepwise backward selection of the predictors [99]. In order to test for possible overfitting problems and to validate the results, we used leave-on-out cross-validation [100] The goodness of fit of each model was assessed as explanatory (*R^2^*_e_) and predictive power (*R^2^*_p_) after cross-validation [101]. The cross-validated models were also evaluated for their accuracy and precision by normalized mean absolute error (nMAE) [102] as well as concordance correlation coefficient (CCC) [103]. The presence of non-linear patterns in the model residuals was tested by fitting a generalized additive model (GAM) [104] between the model residuals and the covariates, respectively [101]. All statistical analyses were performed in R 3.6.0 [105].

## Figures and Tables

**Table 1:** Data for the classical morphometric variables used in the principal component analysis (PCA). Values are reported as mean ± standard error of the mean per population and per species, whereas the number in brackets is the sample size (i.e., number of individuals per population or species). Values that do not share a letter are significantly different (*p* < 0.05).

Population		Leaves	Calyces		Outer Bracts	Inner Bracts	
ID	Species	LMA (g/m^2^)	Tube Length (mm)	Teeth Length (mm)	Area (mm^2^)	Length (mm)	Area (mm^2^)
C-VA	*G. cordifolia*	182.24 ± 11.25 (9) bc	1.56 ± 0.04 (9) bc	2.33 ± 0.11 (9) a	2.73 ± 0.53 (5) a	3.62 ± 0.18 (7) ab	2.49 ± 0.32 (7) ab
C-FZ	*G. cordifolia*	187.82 ± 12.93 (11) bc	1.85 ± 0.04 (11) a	1.92 ± 0.07 (11) bc	4.67 ± 0.50 (5) a	4.08 ± 0.17 (9) ab	2.95 ± 0.17 (9) ab
M-GP	*G. meridionalis*	257.86 ± 9.70 (15) a	1.74 ± 0.04 (15) ab	2.00 ± 0.03 (15) abc	4.44 ± 0.14 (7) a	4.2 ± 0.09 (15) a	2.92 ± 0.14 (15) ab
M-MU	*G. meridionalis*	216.41 ± 9.59 (11) ab	1.47 ± 0.07 (6) c	1.97 ± 0.11 (6) abc	4.09 ± 0.24 (6) a	4.13 ± 0.18 (5) ab	2.80 ± 0.13 (5) ab
M-SF	*G. meridionalis*	217.07 ± 6.95 (9) ab	1.68 ± 0.08 (6) abc	1.85 ± 0.04 (6) bc	3.69 ± 0.77 (4) a	3.73 ± 0.17 (7) ab	2.88 ± 0.22 (7) ab
M-FV	*G. meridionalis*	214.26 ± 11.76 (9) ab	1.57 ± 0.04 (9) bc	2.00 ± 0.07 (9) abc	2.97 ± 0.28 (3) a	4.13 ± 0.18 (8) ab	2.18 ± 0.16 (8) b
M-CI	*G. meridionalis*	188.65 ± 8.67 (11) bc	1.74 ± 0.06 (11) ab	2.06 ± 0.08 (11) abc	3.61 ± 0.25 (5) a	4.14 ± 0.15 (10) ab	3.07 ± 0.21 (10) ab
M-CS	*G. meridionalis*	184.09 ± 8.29 (15) bc	1.85 ± 0.06 (15) a	1.82 ± 0.06 (15) c	4.46 ± 0.31 (7) a	4.09 ± 0.18 (13) ab	2.99 ± 0.22 (13) ab
M-FI	*G. meridionalis*	176.49 ± 5.64 (11) bc	1.98 ± 0.12 (11) a	2.02 ± 0.1 (11) abc	4.38 ± 0.55 (6) a	3.45 ± 0.23 (10) b	2.45 ± 0.32 (10) ab
N-MO	*G. neapolitana*	169.90 ± 5.88 (15) c	1.81 ± 0.04 (15) a	2.14 ± 0.06 (15) ab	3.72 ± 0.32 (9) a	4.03 ± 0.26 (9) ab	3.41 ± 0.31 (9) a
All	*G. cordifolia*	185.32 ± 8.53 (20) b	1.72 ± 0.04 (20) a	2.10 ± 0.07 (20) a	3.70 ± 0.47 (10) a	3.88 ± 0.13 (16) a	2.75 ± 0.17 (16) a
All	*G. meridionalis*	208.74 ± 4.51 (81) a	1.75 ± 0.03 (73) a	1.96 ± 0.03 (73) a	4.07 ± 0.15 (38) a	4.00 ± 0.07 (68) a	2.79 ± 0.08 (68) a
All	*G. neapolitana*	169.90 ± 5.88 (15) b	1.81 ± 0.04 (15) a	2.14 ± 0.06 (15) a	3.72 ± 0.32 (9) a	4.03 ± 0.26 (9) a	3.41 ± 0.31 (9) a

**Table 2:** Results of the multiple regression models using classical and geometric morphometrics PCs as dependent variables and environmental variables as predictors. *R^2^*_e_/*R^2^*_p_ represent explanatory and predictive power, respectively, whereas CCC/nMAE represent concordance correlation coefficient and normalized mean absolute error, respectively. Symbols represent * *p* < 0.05 and ** *p* < 0.01.

Predictors	Dependent Variables			
	PC1 Classical Morphometrics	PC2 Classical Morphometrics	PC1 Geometric Morphometrics	PC2 Geometric Morphometrics
	Estimate (Confidence Interval)	Estimate (Confidence Interval	Estimate (Confidence Interval	Estimate (Confidence Interval)
(Intercept)	18.70 (7.91–29.50) *	7.16 (4.31–10.02) **	1.20 (0.49–1.91) *	−0.01 (−0.03–0.01)
Solar radiation	−1.10 × 10^−3^ (−1.80 × 10^−3^ to −4.00 × 10^−4^) *			
Annual precipitation	−3.60 × 10^−3^ (−5.60 × 10^−3^ to −1.30 × 10^−3^) *			
Temperature seasonality		−1.18 × 10^−3^ (−1.65 × 10^−3^ to −7.10 × 10^−4^) **	−2.0 × 10^−4^ (−3.10 × 10^−4^ to −8.00 × 10^−5^) *	
Mean Temperature driest quarter				8.00 × 10^−5^ (−8.00 × 10^−5^ to 1.90 × 10^−4^)
*R^2^*_e_/*R^2^*_p_	0.697/0.545	0.760/0.617	0.580/0.417	0.179/0.000
CCC/nMAE	0.683/0.212	0.782/0.144	0.624/0.229	0.000/0.377

**Table 3:** Sampled populations, with their ID, name, region, and country. Populations are sorted according to species and latitude, from north to south. The identification of each species was performed according to the morphological description and distribution reported by [2,3]. Altitude is given as m above sea level, whereas geographic coordinates (latitude and longitude) are in decimal degrees (WGS-84).

ID	Description	Species	Date of Collection	Elevation	Latitude	Longitude
C-VA	Val de Rhêmes, Vallée d’Aoste, Italy	*G. cordifolia*	13 July 2017	2428	45.51	7.08
C-FZ	Campocecina-Fivizzano, Toscana, Italy	*G. cordifolia*	24 July 2017	1293	44.11	10.15
M-GP	Grobničko polje, Primorje-Gorski kotar, Croatia	*G. meridionalis*	8 October 2017	308	45.38	14.51
M-MU	Mala Učka, Primorje-Gorski kotar, Croatia	*G. meridionalis*	8 October 2017	926	45.30	14.19
M-SF	Monte S. Franco, Abruzzo, Italy	*G. meridionalis*	1 August 2017	1647	42.46	13.41
M-FV	Fonte Vedice, Abruzzo, Italy	*G. meridionalis*	24 October 2017	1080	42.34	13.58
M-CI	Monte Cigno, Campania, Italy	*G. meridionalis*	8 October 2017	360	41.30	14.54
M-CS	Camposauro, Campania, Italy	*G. meridionalis*	25 June 2017	1182	41.18	14.60
M-FI	Timpa del Principe-Frascineto, Calabria, Italy	*G. meridionalis*	8 August 2017	1668	39.87	16.27
N-MO	Monte S. Angelo a Tre Pizzi, Campania, Italy	*G. neapolitana*	16 July 2017	1385	40.65	14.50

## Supplementary Materials

The following are available online at https://www.mdpi.com/2223-7747/9/3/314/s1, **Supplement 1:** All classical morphometric measurements for the studied populations. Values are mean ± standard error of the mean. **Supplement 2:** Complete imagery and data for classical and geometric morphometric analyses. **Supplement 3:** PC2 vs. PC3 for classical morphometric data. **Supplement 4:** Scatterplots between geometric morphometric PC2 scores and mean temperature of driest quarter. **Supplement 5:** High resolution digital voucher specimens for all sampled populations.
