# Supplementary material for: Explaining Intricate Morphometric Variability with Environmental Predictors: The Case of Globularia cordifolia Species Complex
Source: Plants (Basel). 2020 Mar 3;9(3):314. doi: 10.3390/plants9030314 (PMC7154870; doi:10.3390/plants9030314)
Supplement: Supplementary file 1 [file plants-09-00314-s001.zip › Supplement 1.docx]

Supplement 1: All classical morphometrics measurements for the studied populations. Values are mean ± standard error of the mean.

|  |  | **Leaves** | | | | | |
| --- | --- | --- | --- | --- | --- | --- | --- |
| **Species** | **ID** | **Area (mm^2^)** | **Length (mm)** | **Width (mm)** | **Weight (mg)** | **L:W** | **LMA (g/m^2^)** |
| *G. cordifolia* | C-VA | 37.77 ± 1.72 (9) | 14.66 ± 0.34 (9) | 4.97 ± 0.21 (9) | 6.25 ± 0.4 (9) | 3.01 ± 0.13 (9) | 182.24 ± 11.25 (9) |
| *G. meridionalis* | M-GP | 46.96 ± 2.83 (15) | 19.43 ± 0.63 (15) | 5.49 ± 0.24 (15) | 11.76 ± 0.78 (15) | 3.6 ± 0.15 (15) | 257.86 ± 9.70 (15) |
| *G. meridionalis* | M-MU | 52.17 ± 5.29 (11) | 20.5 ± 1.43 (11) | 5.3 ± 0.38 (11) | 11.22 ± 1.19 (11) | 3.94 ± 0.26 (11) | 216.41 ± 9.59 (11) |
| *G. cordifolia* | C-FZ | 21.14 ± 1.49 (11) | 12.25 ± 0.78 (11) | 3.73 ± 0.14 (11) | 4.48 ± 0.29 (11) | 3.43 ± 0.16 (11) | 187.82 ± 12.93 (11) |
| *G. meridionalis* | M-SF | 26.92 ± 1.68 (9) | 14.00 ± 0.66 (9) | 3.95 ± 0.17 (9) | 5.55 ± 0.45 (9) | 3.61 ± 0.18 (9) | 217.07 ± 6.95 (9) |
| *G. meridionalis* | M-FV | 32.79 ± 2.78 (9) | 16.28 ± 0.66 (9) | 4.24 ± 0.27 (9) | 7.25 ± 1.06 (9) | 3.94 ± 0.23 (9) | 214.26 ± 11.76 (9) |
| *G. meridionalis* | M-CI | 52.79 ± 2.92 (11) | 19.78 ± 0.81 (11) | 6.09 ± 0.2 (11) | 9.98 ± 0.74 (11) | 3.27 ± 0.08 (11) | 188.65 ± 8.67 (11) |
| *G. meridionalis* | M-CS | 33.12 ± 4.51 (15) | 13.36 ± 0.97 (15) | 4.26 ± 0.33 (15) | 5.70 ± 0.66 (15) | 3.17 ± 0.07 (15) | 184.09 ± 8.29 (15) |
| *G. neapolitana* | N-MO | 29.17 ± 2.64 (15) | 12.39 ± 0.47 (15) | 4.51 ± 0.22 (15) | 4.75 ± 0.35 (15) | 2.85 ± 0.13 (15) | 169.90 ± 5.88 (15) |
| *G. meridionalis* | M-FI | 33.7 ± 2.62 (11) | 13.63 ± 0.77 (11) | 4.89 ± 0.25 (11) | 5.89 ± 0.45 (11) | 2.84 ± 0.10 (11) | 176.49 ± 5.64 (11) |
|  |  |  |  |  |  |  |  |
|  |  | **Calyces** | | |  |  |  |
| **Species** | **ID** | **Tube Length (mm)** | **Teeth Length (mm)** | **Teeth:Tube** |  |  |  |
| *G. cordifolia* | C-VA | 1.56 ± 0.04 (9) | 2.33 ± 0.11 (9) | 1.5 ± 0.07 (9) |  |  |  |
| *G. meridionalis* | M-GP | 1.74 ± 0.04 (15) | 2.00 ± 0.03 (15) | 0.87 ± 0.02 (15) |  |  |  |
| *G. meridionalis* | M-MU | 1.47 ± 0.07 (6) | 1.97 ± 0.11 (6) | 0.76 ± 0.02 (6) |  |  |  |
| *G. cordifolia* | C-FZ | 1.85 ± 0.04 (11) | 1.92 ± 0.07 (11) | 1.04 ± 0.03 (11) |  |  |  |
| *G. meridionalis* | M-SF | 1.68 ± 0.08 (6) | 1.85 ± 0.04 (6) | 0.92 ± 0.04 (6) |  |  |  |
| *G. meridionalis* | M-FV | 1.57 ± 0.04 (9) | 2.00 ± 0.07 (9) | 0.79 ± 0.02 (9) |  |  |  |
| *G. meridionalis* | M-CI | 1.74 ± 0.06 (11) | 2.06 ± 0.08 (11) | 0.86 ± 0.03 (11) |  |  |  |
| *G. meridionalis* | M-CS | 1.85 ± 0.06 (15) | 1.82 ± 0.06 (15) | 1.00 ± 0.04 (15) |  |  |  |
| *G. neapolitana* | N-MO | 1.81 ± 0.04 (15) | 2.14 ± 0.06 (15) | 1.19 ± 0.03 (15) |  |  |  |
| *G. meridionalis* | M-FI | 1.98 ± 0.12 (11) | 2.02 ± 0.10 (11) | 1.03 ± 0.03 (11) |  |  |  |
|  |  |  |  |  |  |  |  |
|  |  | **Outer Bracts** | | | |  |  |
| **Species** | **ID** | **Area (mm^2^)** | **Length (mm)** | **Width (mm)** | **L:W** |  |  |
| *G. cordifolia* | C-VA | 2.73 ± 0.53 (5) | 3.14 ± 0.28 (5) | 1.47 ± 0.28 (5) | 2.43 ± 0.40 (5) |  |  |
| *G. meridionalis* | M-GP | 4.44 ± 0.14 (7) | 4.31 ± 0.17 (7) | 1.65 ± 0.09 (7) | 2.66 ± 0.16 (7) |  |  |
| *G. meridionalis* | M-MU | 4.09 ± 0.24 (6) | 4.45 ± 0.20 (6) | 1.48 ± 0.04 (6) | 3.05 ± 0.17 (6) |  |  |
| *G. cordifolia* | C-FZ | 4.67 ± 0.50 (5) | 3.93 ± 0.40 (5) | 1.71 ± 0.10 (5) | 2.28 ± 0.11 (5) |  |  |
| *G. meridionalis* | M-SF | 3.69 ± 0.77 (4) | 3.45 ± 0.30 (4) | 2.08 ± 0.18 (4) | 1.67 ± 0.12 (4) |  |  |
| *G. meridionalis* | M-FV | 2.97 ± 0.28 (3) | 3.51 ± 0.56 (3) | 1.42 ± 0.11 (3) | 2.55 ± 0.54 (3) |  |  |
| *G. meridionalis* | M-CI | 3.61 ± 0.25 (5) | 3.59 ± 0.18 (5) | 1.60 ± 0.07 (5) | 2.28 ± 0.07 (5) |  |  |
| *G. meridionalis* | M-CS | 4.46 ± 0.31 (7) | 3.76 ± 0.13 (7) | 1.82 ± 0.10 (7) | 2.10 ± 0.08 (7) |  |  |
| *G. neapolitana* | N-MO | 3.72 ± 0.32 (9) | 3.44 ± 0.29 (9) | 1.72 ± 0.11 (9) | 2.01 ± 0.14 (9) |  |  |
| *G. meridionalis* | M-FI | 4.38 ± 0.55 (6) | 3.44 ± 0.26 (6) | 1.83 ± 0.11 (6) | 1.88 ± 0.10 (6) |  |  |

|  |  |  |  |  |  |  |  |
| --- | --- | --- | --- | --- | --- | --- | --- |
|  |  | **Inner Bracts** | | | |  |  |
| **Species** | **ID** | **Area (mm^2^)** | **Length (mm)** | **Width (mm)** | **L:W** |  |  |
| *G. cordifolia* | C-VA | 2.49 ± 0.32 (7) | 3.62 ± 0.18 (7) | 1.19 ± 0.15 (7) | 3.51 ± 0.5 (7) |  |  |
| *G. meridionalis* | M-GP | 2.92 ± 0.14 (15) | 4.2 ± 0.09 (15) | 1.08 ± 0.04 (15) | 4.07 ± 0.14 (15) |  |  |
| *G. meridionalis* | M-MU | 2.80 ± 0.13 (5) | 4.13 ± 0.18 (5) | 1.01 ± 0.1 (5) | 4.27 ± 0.35 (5) |  |  |
| *G. cordifolia* | C-FZ | 2.95 ± 0.17 (9) | 4.08 ± 0.17 (9) | 1.20 ± 0.06 (9) | 3.53 ± 0.25 (9) |  |  |
| *G. meridionalis* | M-SF | 2.88 ± 0.22 (7) | 3.73 ± 0.17 (7) | 1.22 ± 0.08 (7) | 3.17 ± 0.15 (7) |  |  |
| *G. meridionalis* | M-FV | 2.18 ± 0.16 (8) | 4.13 ± 0.18 (8) | 0.83 ± 0.05 (8) | 5.28 ± 0.38 (8) |  |  |
| *G. meridionalis* | M-CI | 3.07 ± 0.21 (10) | 4.14 ± 0.15 (10) | 1.23 ± 0.07 (10) | 3.51 ± 0.24 (10) |  |  |
| *G. meridionalis* | M-CS | 2.99 ± 0.22 (13) | 4.09 ± 0.18 (13) | 1.24 ± 0.05 (13) | 3.41 ± 0.15 (13) |  |  |
| *G. neapolitana* | N-MO | 3.41 ± 0.31 (9) | 4.03 ± 0.26 (9) | 1.36 ± 0.08 (9) | 3.00 ± 0.17 (9) |  |  |
| *G. meridionalis* | M-FI | 2.45 ± 0.32 (10) | 3.45 ± 0.23 (10) | 1.14 ± 0.07 (10) | 3.25 ± 0.33 (10) |  |  |
|  |  |  |  |  |  |  |  |
